# Cryopreservation of vegetative thalli of *Ulva* species

**DOI:** 10.1007/s10811-024-03300-3

**Published:** 2024-07-06

**Authors:** Clara Simon, Antoine Fort, Ronan Sulpice

**Affiliations:** 1 Plant Systems Biology Lab, School of Biological & Chemical Sciences, Ryan Institute & Marei centre for Marine, University of Galway, Galway, Ireland; 2 Dept. of Bioveterinary and Microbial Sciences, Technological University of The Shannon: Midlands, Athlone, Co. Roscommon Ireland

**Keywords:** *Ulva*, Chlorophyceae, Cryopreservation, Biobanking, Aquaculture

## Abstract

Sea lettuce (*Ulva*) is a genus of green macroalgae present along all the coasts of the world's oceans. It represents about 100 species with diverse habitats. Inter- and intra-species natural variation is very large, both in terms of growth characteristics and biomass biochemical composition. As a result, *Ulva* biomass has a wide range of applications and strain selection can achieve significant increases in yield(s). Establishing solid, long term and cost-effective methodologies for the conservation of *Ulva* genetic diversity is then required to safeguard and reuse selected strains. Here, we report a cryopreservation-based protocol for the long-term preservation of foliose *Ulva* strains. Strains from seven different *Ulva* species were cryopreserved for 15 and/or 120 days in liquid nitrogen, and of the 3 replicates cryopreserved, at least one survived, allowing us to successfully recover all strains. On average, among all specimen cryo-preserved, 82% of them survived and grew post cryo-preservation.

**Supplementary information:**

The online version contains supplementary material available at 10.1007/s10811-024-03300-3.

## Introduction

The green macroalga *Ulva* represents a source of biomass that can be harnessed in large quantities in various ecosystems around the world. *Ulva* biomass is increasingly being valorized and cultivated as it offers a valuable chemical content often associated with high growth rate and a broad tolerance to different environmental conditions (Fort et al. 2020). These characteristics make *Ulva* a target for a wide range of applications, including pharmaceuticals, bioremediation, food and feed (Simon et al. 2022).

However, *Ulva* aquaculture is still in its infancy and its development faces many challenges (Simon et al. 2022). The lack of long-term methodologies for maintaining and conserving cultivated *Ulva* stocks is one of the many obstacles to the development of *Ulva* aquaculture. While preservation techniques, such as lyophilization, freezing and cryopreservation, of microalgal cultures is relatively well documented and implemented, much remains to be done when it comes to the preservation of the vegetative tissues of macroalgae (Foo et al. 2023). Macroalgae are usually kept alive over long term and biobanking methods have been well established, e.g., for brown macroalgae (Kono et al. 1998; Zhang et al. 2007a, b; Nanba et al. 2009; Heesch et al. 2012; Visch et al. 2019). If successfully implemented, cryopreservation would offer the possibility of storing and preserving biological specimens over long periods, with little labor involved, guaranteeing a contamination-free environment and genetic stability (Morris 1981).

Some studies have investigated the use of cryopreservation for single cell life stage of different macroalgae (Day et al. 1997; Kono et al. 1997; Zhang et al. 2007a, b; Heesch et al. 2012; Visch et al. 2019) and few studies have investigated the cryopreservation of vegetative tissue thalli, e.g., for *Gracilaria corticata, Hypnea musiformis, Ulva intestinalis, Ulva lactuca* and *Ulva prolifera* (Kono et al. 1997; Lalrinsanga et al. 2009; Lee & Nam 2016)*.* The possibility of preserving vegetative tissues instead of single cells (spores or gametes) offers some advantages such as a very rapid regeneration, a reduction of the risk of microbiome loss and there is no requirement for the control of the life cycle of the macroalgae of interest, which can be a major issue.

We investigated the effect of pre-cryopreservation and recovery culture conditions, prefreezing incubation time and regenerative efficacy of cryopreserved thalli after short- (15 days) and long- (120 days) term storage for 7 different foliose *Ulva* species. We report the most effective cryopreservation protocol of *Ulva* foliose vegetative strains that we obtained. A laboratory protocol for cyopreservation and recovery of *Ulva *vegetative thalli developed during this study is available in Supplementary File S1.

## Material and methods

### Seaweed material and stock culture conditions

18 intertidal foliose *Ulva* samples were collected at various sampling sites in Galway Bay (Ireland), Brittany (France) and Portugal (Ilhavo) (Supplementary Table S2). Immediately after collection samples were placed in bags filled with seawater and brought back to the laboratory in a coolbox. Samples collected outside Ireland were shipped in individual Falcon tubes filled with seawater inside coolboxes. Upon arrival in the laboratory, the tissue samples were wiped with paper tissue to remove possible epiphytes and rinsed with artificial seawater. The holdfast was removed to ensure better homogeneity of the tissue, and the remaining thalli were placed in 500 mL glass bottles filled with media containing artificial seawater (ASW Salinity 35 ppt; 37 g L^-1^; Red Sea Coral Pro) and 1X Cell-HI F2P nutrients (Varicon Aqua; [N]=[P]=1 mM), at a constant temperature of 15±1°C under fluorescent light (Osram T5 tubes) at an intensity of 200 μmol photons m^-2^s^-1^ and a light:dark photoperiod of 12h:12h. Seawater was changed every week and the individuals were kept in those conditions for at least 3 months prior to experiments to ensure the acclimation of all strains to the growth conditions. The species identification of the individual collected was performed using the Cleaved Amplified Polymorphic Sequences (CAPS) assay described in Fort et al., (2021) from DNA obtained using magnetic beads (Fort et al. 2018).

### Cryopreservation setup

Prior to cryopreservation, the thalli of each strain were cut into 3 discs of 113 mm^2^ and the 3 discs were placed into a 2 mL microtube containing 750 µL artificial seawater (ASW) buffered with 0.01 M N-2-hydroxyethylpiperazine-N-2-ethanesulfonic acid (HEPES) at pH 8.0. Each strain/treatment combination tested was repeated 2 to 3 times.

Glycerol was used as the cryoprotective additive at 40% glycerol, dissolved in sterilized ASW and buffered with 0.01 M HEPES, pH 8.0. The cryoprotective additive was added slowly (in 15 min) to a final volume equal to the volume of vegetative thalli suspension (750 µL) in a cryogenic vial (2 mL), for a final concentration of 20% glycerol. After addition of the cryoprotectant, the vial was left for 45 min of equilibration time at room temperature.

### Freezing and thawing procedure

After the addition of the cryoprotectant, the vials were placed in a Mr. Frosty Freezing container (Thermo Scientific 5100-0001) filled with isopropanol and transferred to a -80 °C freezer to achieve a cooling rate of ~1 °C min^−1^. The vials were kept in -80 °C for 1 or 2 h. As the starting temperature was 20°C, the vials kept one hour at -80°C reached -40°C before being transferred to liquid nitrogen (LN, -196°C). The vials kept for 2 h reached -80°C after 1h 40min, so were transferred to LN when they were at -80°C. The vials in LN were stored in cryostore boxes for a period of 15 days or 120 days.

After LN treatment, the vials were thawed in a dry bath incubator at 40 °C until the samples had completely melted. In order to remove cryoprotectants, the thawed thalli were then transferred to 30 mL pre-cooled (4°C) artificial seawater 0.01M HEPES pH 8 for 30 min.

### Recovery procedure

The vegetative thalli post-cryopreservation were then transferred to petri dishes containing 50 mL of ASW buffered with 0.01M HEPES pH 8 at a constant temperature of 15±1°C under fluorescent lights with an intensity of 80 μmol photons m^-2^s^-1^ and a light:dark photoperiod of 12h:12h. Next day (after 24 h), nutrients were added to the medium in the form of 0.5× Cell-HI F2P (Varicon Aqua; [N]=[P]=0.5 mM).

After one week, the thalli were transferred into conical flasks containing 100 mL artificial seawater and 0.5× Cell-HI F2P with bubbling, at a constant temperature of 15±1°C under fluorescent light with an increased light intensity of 150 μmol photons m^-2^s^-1^ and a light:dark photoperiod of 12h:12h. The medium was replaced every 3 days during the first month of post-recovery.

### Viability assessment

After thawing, the viability and survival rate of vegetative thalli was assessed by daily visual evaluation of thallus colour and growth. After 10 days of recovery, an image of each strain was taken, and image analysis with Image J software (https://imagej.nih.gov/ij/) was used to determine the percentage of white tissue, corresponding to dead tissue. After one month, when an increase in surface area was observed, the strain was considered to have successfully recovered, whilst when no visible growth was observed, the strains were considered as not having recovered.

### Statistical analysis

All statistical analysis were performed using R (R Core Team 2020). Fisher’s exact test with a significance level of p<0.05 was used to assess the independence of categorical data and to evaluate the effect of prefreezing temperature, species (pairwise comparison), and short-term and long-term storage on the recovery success of the tested *Ulva* strains.

## Results

Thallus survival was observed within 7 days after thawing, with dead thalli losing their pigments during this period. None of the thalli which fully lost their pigmentation during this period could recover afterwards. During this first week, we also observed sporulation of some tissues, in whole or in part of the thallus, for 5 *Ulva lacinulata* strains. Those individuals did not recover, most likely due to contamination of the media by microalgae (Supplementary Table S3).

**Fig. 1:**
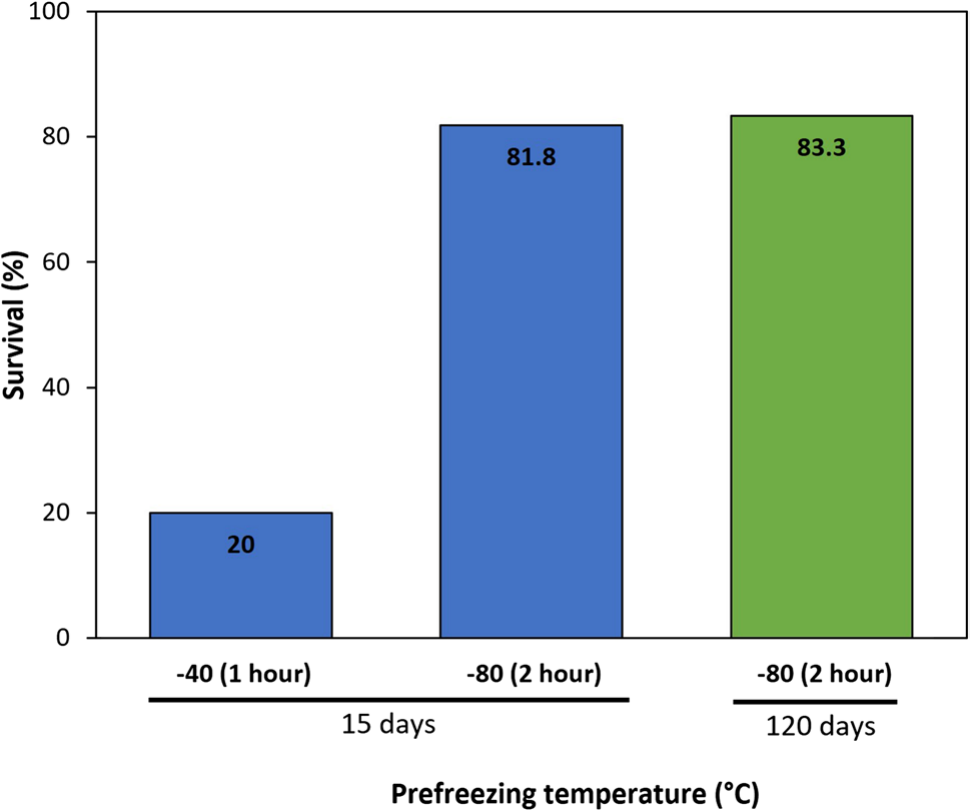
Effect of prefreezing temperatures (-40 and -80 °C) on the survival (%) of *Ulva* thalli and the storage time in liquid nitrogen (15 days and 120 days). -40 °C have been tested on 5 different strains, n=2. -80 °C have been tested for 14 days on 38 strains among 5 different species and for 120 days on 30 strains among 5 different species (Supplementary Table S3). Recovery percentages between the -40 °C treatment and -80 °C treatments were significantly different (Fisher’s exact test for independence between -40 °C and -80 °C treatment, p < 0.05)

Prefreezing temperatures have a critical impact on thallus survival. The survival of thalli pre-frozen at -40°C was around 20%, while thalli pre-frozen at -80°C showed a survival of about 82% (Figure 1)

Short-time (15 days) and long-term (120 days) preservation in liquid nitrogen did not show any differences in term of survival success post cryopreservation, with an average survival of around 82% for the 78 samples tested (among 19 different strains) (Figure 1; Supplementary Table S3). The preservation time therefore does not seem to impact the recovery and viability of the cryopreserved strains.

**Fig. 2:**
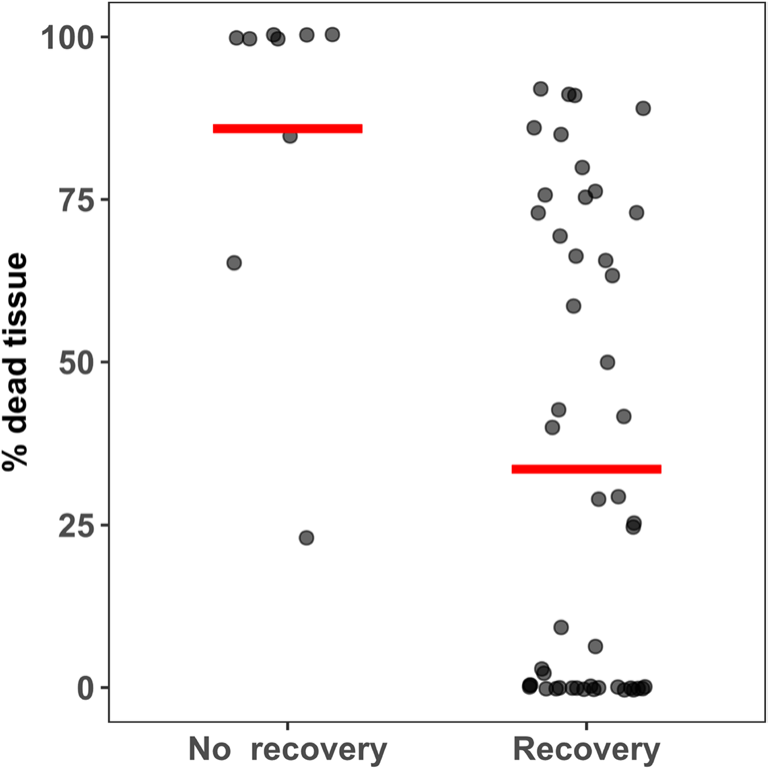
Proportion of dead tissue (%) at Day 10 post-cryopreservation for the strains which have been then identified as fully recovered or not recovered after day 30 post cryopreservation. n strains recovered = 47; n strains not recovered = 9. Red line represents the main percentage of dead tissue per category

On day 10, tissue viability was assessed by identifying the proportion of dead (white) tissue for each strain (Figure 2). After one month of post-cryopreservation cultivation, if the strain was alive and showed an increase in tissue surface, the strain was considered fully recovered. Figure 2 shows that if some of the discs are still green after 10 days, comprising even up to 91% of dead tissue, there was still a possibility of recovery in most of the cases, with only three strains which were not fully white failing to recover. In contrast none of the strains with 100% of the surface white after 10 days recovered.

**Fig. 3:**
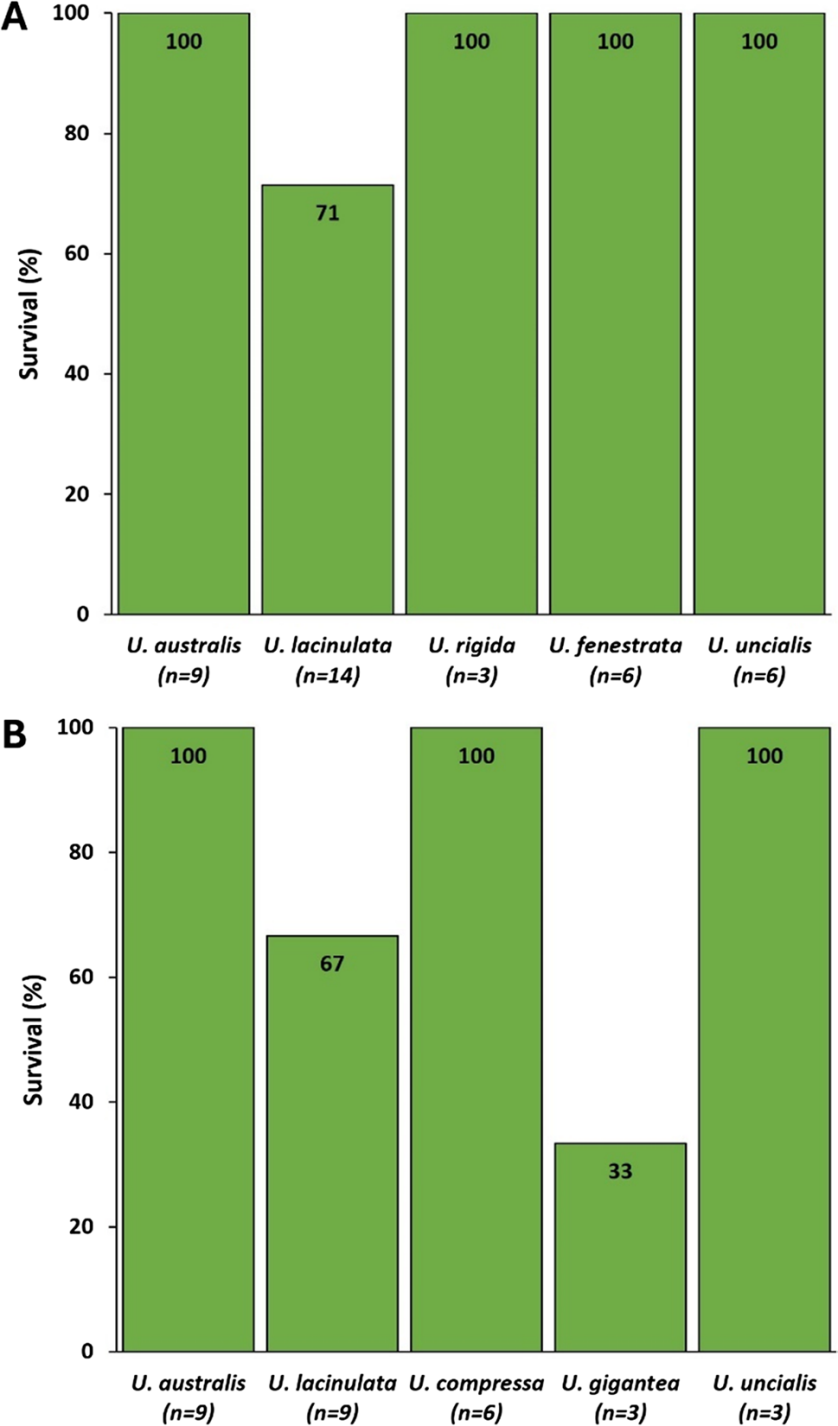
Survival (%) of different *Ulva* species (n= species replicates). **(A)** Survival rate of 5 different *Ulva* foliose species after 14 days of cryopreservation **(B)** Survival rate of 5 different *Ulva* foliose species after 120 days of cryopreservation. Survival percentages between species and between short-and long-term were not significantly different (Fisher’s exact test for independence between -40 °C and -80 °C treatment, p>0.05; Supplementary Table S4)

Variation in survival between *Ulva* species were observed (Figure 3). We tested for significance the variations in surviva between species cryopreserved for short (Figure 3A) or long term (Figure 3B) and no significant differences were identified between species, both for short term and long term cryopreservation (Fisher’s Exact Test, p>0.05; Supplementary Table S4). Variation in survival was then tested between short term and long-term cryopreservation for the common set of species tested, i.e., *U. australis*, *U. lacinulata* and *U. uncialis*, and no significant differences were observed (p>0.05).

**Fig. 4:**
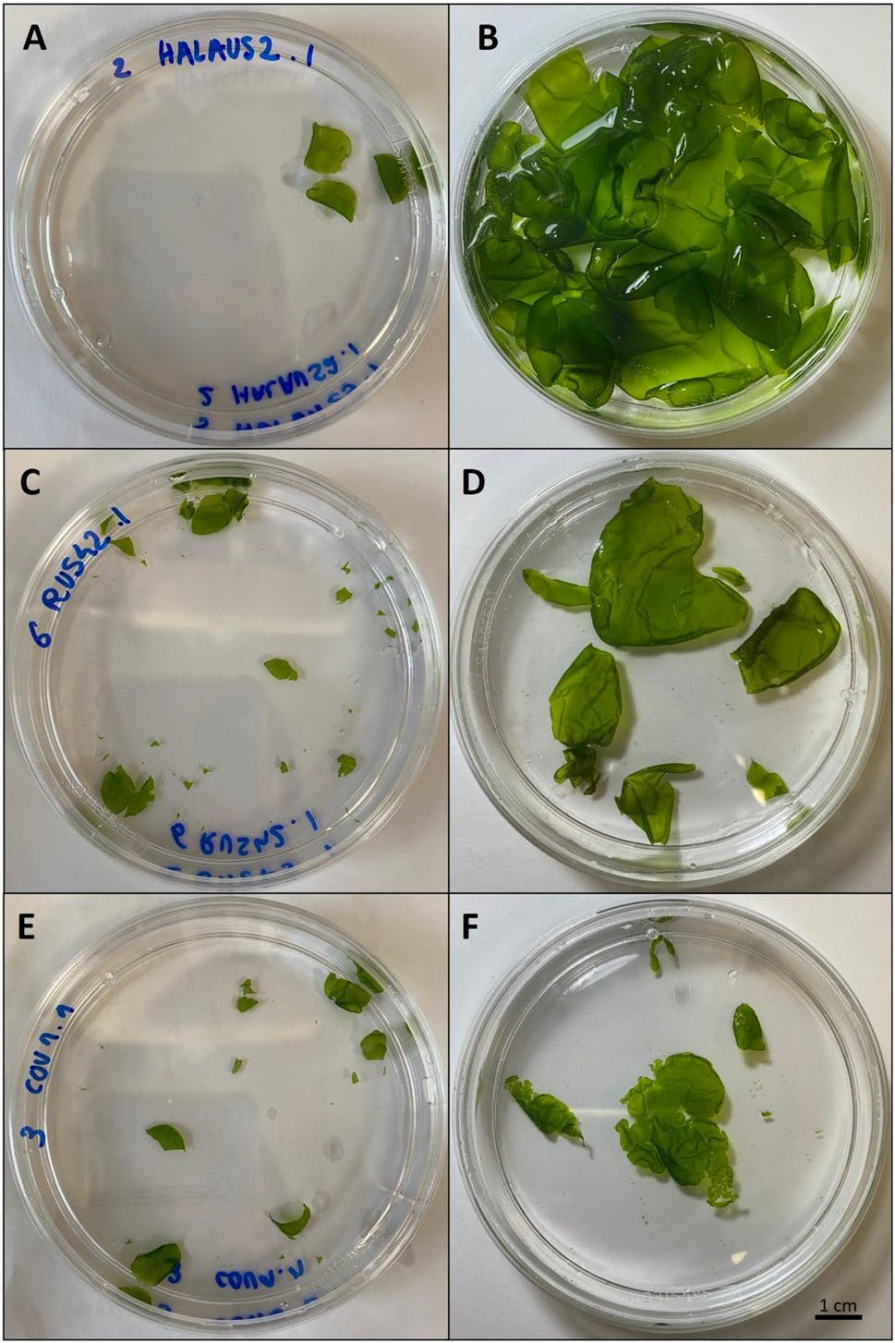
Pictures of 3 different *Ulva* strains after 120 days of cryopreservation, immediately post-thaw (left column) and after one month of recovery (right column; *Ulva australis* just after (A) and one month (B) post cryopreservation; *Ulva compressa* strain just after (C) and one month (D) post cryopreservation; *Ulva lacinulata* strain just after (E) and one month (F) post cryopreservation

The totality of *U. australis*, *U. rigida*, *U. compressa*, *U. unicialis* and *U. fenestrata* species, for a total of 43 strains, recovered well and green color thalli associated with significant regrowth after one month post cryopreservation were observed (Figure 4). In contrast, *U. gigantea* species showed a low survival, around 33%, but only 3 stains were tested, and statistical analysis did not allow to identify significant variation in survival between this species and others. *U. lacinulata,* with 23 individuals from 8 strains tested also showed a relatively low recovery for both long-term and short-term storage, with an average survival around 69%. The 5 *U. lacinulata* strains whose sporulation was detected were also individuals whose contamination by microalgae was observed in pre-culture (Supplementary Table S3). When the strains were thawed and re-cultured after cryopreservation, microalgae grew and could have prevented the strains from developing properly.

Our results indicate an average survival of 82%, but it is important to note that as each strain was in fact successfully recovered (Supplementary Table S3). Indeed, among the 3 replicates of each cryopreserved strain, at least one survived cryopreservation.

## Discussion

Freezing living cells without damaging their ability to recover is a complex biological process that still require optimization for many species, particularly for macroalgae. Important parameters for successful cryopreservation have been identified over the years, since the first cryopreservation trials were documented in the 1960s (Morris 1981). Cooling rate, culture conditions before and after cryopreservation, and the right choice and concentration of cryoprotective additives have been identified as the main parameters for successful cryopreservation (Lalrinsanga et al. 2009).

Cooling rate is a critical parameter for cell viability. Slow cooling rates are necessary to allow sufficient time for the cell to maintain correct osmotic equilibrium. Indeed, during cooling, two major cellular events can occur. The most widely observed is the process of plasmolysis, which results from dehydration of the cell and collapse of the cell wall (Morris 1981). The viability of the cell will then be at risk when thawing the cells, as a damaged cell will lose its elasticity and the protoplast will not swell and remain contracted. In addition, during cooling, ice formation can occur between the cell wall and the protoplast (Taylor & Fletcher 1998). Due to differences in chemical potential between the supercooled water inside the protoplast and the ice on the outside, the protoplast detaches from the cell wall (pseudo-plasmolysis), leading to cell death. It has been shown that the cooling rate at which intracellular ice formation occurs depends on a cell water permeability and surface-to-volume ratio (Morris 1981). Each type of cells has an optimal cooling rate, which is about identifying the correct balance between the two important damaging processes, the decrease in cell dehydration which increases when cooling is fast, and the increase of ice formation with cooling is slow (Zhang et al. 2007a, b). In this study, in accordance with Lee and Nam (2016) work on the tubular monostromatic *U. prolifera*, we found that the optimum cooling time for *Ulva* species is around 1°C min^-1^ until it reaches the temperature of -80°C. The thalli can then be safely transferred to -196°C for long term without damaging cell viability. A preefrezing temperature of -40°C instead of -80°C before direct transfer at -196°C significantly decreased the post-recovery survival rate of *Ulva* species. These results contradict those of Kono et al., (1997) who showed that survival rate was progressively lower at lower temperatures before freezing for the species *U. intestinalis*. However, this observation was made using DMSO as the cryoprotectant, which may explain the difference in survival obtained in this study. Then, at temperatures below -139°C, biophysical processes are strongly reduced (no more ice crystal formation) and genetically stable (Morris 1981), so viability is most likely independent of storage time, in accordance to our findings and Kono et al., (1997).

Culture conditions before and after freezing appear to be also an essential parameter for a successful cryopreservation and high post-thaw survival (Zhang et al. 2007a, b). It was observed in this study that the condition of the starting material prior to cryopreservation is an essential point for successful recovery. The material must be in good state of growth and importantly be devoid of visible bacterial/microalgal contamination. The 5 individuals from the species *U. lacinulata* whose sporulation was detected are also individuals whose contamination by microalgae was observed in pre-culture (Supplementary Table S3). We hypothesize that the presence of microalgae induced a stress to the specimen, leading to sporulation. Sporulation events in *Ulva* species are known to be induced by external stimuli, such as nutrient availability, salinity, temperature (Balar & Mantri 2020). The presence of microalgae can have an impact on nutrient availability by competing for resources with *Ulva*, which can represent a stress for the macroalgae. For this reason, we suggest changing the media frequently, every 3 days, during the first month of recovery to prevent the microalgae from overgrowing *Ulva*. *Ulva gigantea* showed a very low recovery (33%), but only 1 strain was tested, and more strains should be evaluated to draw clear conclusions. The elimination of microalgal contamination should be ensured prior cryopreservation, as this has proved a major problem for strain recovery and ideally cultures should be unialgal and contain only the strain of interest. However, removing microalgal contamination without compromising the essential microbiome associated with *Ulva *remains a challenge and further work should be carried out to address this important point. Lalrinsanga et al., (2009) tested different concentrations of glycerol and cryoprotectant (DMSO and ethylene glycol) on *U. lactuca* survival. The highest recovery (~51%) was obtained with 10% DMSO, which is still a fairly low recovery percentage. When using 15% glycerol as cryoprotectant, they obtained a recovery of ~45%, which is significantly lower than the results presented in this study. This is probably due to the fact that the prefreezing temperature was -40°C, for which we also observed a low survival (20%). Lee and Nam (2016) demonstrated that the highest recovery (~92%) for *U. prolifera* gametophyte thalli cryopreservation was obtained using 20% glycerol, same as for the protocol we used. Altogether, many variables come into play, and it is therefore difficult to compare results available in the literature, but the combination of 20% glycerol and a pre-freezing temperature of -80°C combined with a temperature of 40°C during thawing seems to be the right balance to achieve a high percentage survival for *Ulva* species.

In average, 83% of the samples survived the cryo-preservation treatment, but it is important to note that no strain was lost during cryo-preservation (Supplementary Table S3); only a fraction of the replicates was lost. Hence, if several (4-5) replicates of each strain are cryopreserved, all genetic diversity present in the cryo-bank should be preserved. In average, 34% of the tissues were dead at day 10 post-cryopreservation for the strains which recovered post cryopreservation. Partial tissue loss is therefore considered inevitable during cryopreservation but is not an issue for strain recovery. To conclude, this study describes a successful method for long-term cryopreservation of *Ulva* vegetative thalli from seven distromatic foliose species. The possibility of cryo-preserving vegetative tissues is expected to present a major interest for *Ulva* community, as the control of *Ulva* life cycle is no longer necessary for preserving *Ulva* genetic diversity on long term.

## Electronic supplementary material

Below is the link to the electronic supplementary material.

Supplementary file1 (ODT 11 KB)

Supplementary file2 (XLSX 11 KB)

Supplementary file3 (XLSX 21 KB)

Supplementary file4 (XLSX 10 KB)

## Data Availability

Data will be made available on request.
