# Supplementary material for: Cryopreservation of vegetative thalli of Ulva species
Source: J Appl Phycol. 2024 Jul 6;36(5):3011–6. doi: 10.1007/s10811-024-03300-3 (PMC11452468; doi:10.1007/s10811-024-03300-3)
Supplement: Supplementary file 1 [file 10811_2024_3300_MOESM1_ESM.odt]

Laboratory protocol for cryopreservation and recovery of Ulva vegetative thalli

Solutions:

- Artificial seawater (ASW 35ppt; 37 g.L-1; Red Sea Coral Pro) made in 0.01M HEPES (N-2-hydroxyethylpiperazine-′-2-ethanesulfonic acid, C10H14KNO3S, Sigma Aldrich) pH 8  

- Cell-HI F2P nutrients (Varicon aqua)

- 40% glycerol stock (HOCH2CH(OH)CH2OH, ≥99.5%, Sigma Aldrich) in artificial seawater made in 0.01M HEPES pH 8

- Isopropanol(C3H8O, 99.5+%, pure, Fisher Chemical™)

Equipment:

- 2 mL microtube

- MrFrosty (Thermo Scientific™)

- Conventional -80°C freezer

- Liquid nitrogen cryogenic dewar with box for storing in vials

Cryopreservation:

- Cut Ulva tissue in discs of ~3 cm diameter. 

- Place discs in 2 mL microtube.

- Add 750 µL ASW 0.01M HEPES pH8.

- Slowly add 750 µL 40% glycerol stock (50 µL or 100 µL each time up to 750 µL, shaking gently between additions, typically over 15 min), for a final concentration of 20% glycerol.

- Incubate at room temperature for 45 min.

- Place samples in MrFrosty box filled with Isopropanol (to achieve cooling rate of -1°C/min). 

- Place MrFrosty in -80°C freezer for 2 h.

- After 2 h, place microtubes in liquid nitrogen and store them in liquid nitrogen container. The thalli are now cryopreserved. 

Recovery:

- Quickly thaw samples at 40°C in dry bath for ~1-2 min until ice is gone.

- Transfer and incubate tissue in 50 mL falcon tubes containing 30 mL pre-cooled (4°C) ASW 0.01M HEPES pH8 for 30 min. 

- Place tissue in 50 mL petri-dishes filled with 0.01M HEPES pH8 in growth room, light intensity ~80 µmoles and after 24 hours add nutrients (0.5X Cell-HI F2P). 

- After the first week of recovery, replace media with normal ASW (no need for buffering) + nutrients and transfer samples in 100mL conical flasks, add bubbling, place under higher light if possible (~150 µmoles/m2/s).

- Maintain in that stage for two weeks, replacing media every 3 days.

- After the end of the third week post-recovery, samples should be totally recovered.
